# An upper body garment with integrated sensors for people with neurological disorders – early development and evaluation

**DOI:** 10.1186/s42490-019-0002-3

**Published:** 2019-01-30

**Authors:** Margit Alt Murphy, Filip Bergquist, Bengt Hagström, Niina Hernández, Dongni Johansson, Fredrik Ohlsson, Leif Sandsjö, Jan Wipenmyr, Kristina Malmgren

**Affiliations:** 1 Department of Clinical Neuroscience, Institute of Neuroscience and Physiology, Sahlgrenska Academy, University of Gothenburg, Per Dubbsgatan 14, 3rd Floor, SE-41345 Gothenburg, Sweden; 2 Department of Pharmacology, Institute of Neuroscience and Physiology, Sahlgrenska Academy, University of Gothenburg, Gothenburg, Sweden; 3 Department of Materials, Swerea IVF, Mölndal, Sweden; 4 Department of Industrial and Materials Science, Chalmers University of Technology, Gothenburg, Sweden; 5 Swedish School of Textiles, University of Borås, Borås, Sweden; 6 RISE Acreo, Gothenburg, Sweden; 7 MedTech West/Faculty of Caring Science, Work Life and Social Welfare, University of Borås, Borås, Sweden; 8 Department of Industrial and Materials Science, Division of Design & Human Factors, Chalmers University of Technology, Gothenburg, Sweden; 9 Department of Neurology, Sahlgrenska University Hospital, Gothenburg, Sweden

**Keywords:** Wearable technology, Ambulatory monitoring, Neurological disorders, Patient preference, Neurological diagnostic technic, Textiles, Biomedical technology assessment, Accelerometry

## Abstract

**Background:**

In neurology and rehabilitation the primary interest for using wearables is to supplement traditional patient assessment and monitoring in hospital settings with continuous data collection at home and in community settings. The aim of this project was to develop a novel wearable garment with integrated sensors designed for continuous monitoring of physiological and movement related variables to evaluate progression, tailor treatments and improve diagnosis in epilepsy, Parkinson’s disease and stroke. In this paper the early development and evaluation of a prototype designed to monitor movements and heart rate is described. An iterative development process and evaluation of an upper body garment with integrated sensors included: identification of user needs, specification of technical and garment requirements, garment development and production as well as evaluation of garment design, functionality and usability. The project is a multidisciplinary collaboration with experts from medical, engineering, textile, and material science within the wearITmed consortium. The work was organized in regular meetings, task groups and hands-on workshops. User needs were identified using results from a mixed-methods systematic review, a focus group study and expert groups. Usability was evaluated in 19 individuals (13 controls, 6 patients with Parkinson’s disease) using semi-structured interviews and qualitative content analysis.

**Results:**

The garment was well accepted by the users regarding design and comfort, although the users were cautious about the technology and suggested improvements. All electronic components passed a washability test. The most robust data was obtained from accelerometer and gyroscope sensors while the electrodes for heart rate registration were sensitive to motion artefacts. The algorithm development within the wearITmed consortium has shown promising results.

**Conclusions:**

The prototype was accepted by the users. Technical improvements are needed, but preliminary data indicate that the garment has potential to be used as a tool for diagnosis and treatment selection and could provide added value for monitoring seizures in epilepsy, fluctuations in PD and activity levels in stroke. Future work aims to improve the prototype further, develop algorithms, and evaluate the functionality and usability in targeted patient groups. The potential of incorporating blood pressure and heart-rate variability monitoring will also be explored.

## Background

Wearable technology has become increasingly popular in clinical research over the last decade. The primary driver for using wearables in neurology and rehabilitation has been the prospect to supplement patient assessment and monitoring in hospital settings with continuous data collection at home and in community settings. The common understanding is that the use of wearables in clinical applications has a potential to improve diagnosis and to allow continuous monitoring of disease development and thereby individualize treatment [1, 2].

Neurological conditions, such as stroke, Parkinson’s disease (PD) and epilepsy, are major causes of disability worldwide [3–5]. With an aging population and an increased longevity, the prevalence of neurological disorders is expected to increase in the future [6, 7]. Neurological disorders represent the largest cause of disability adjusted life years [5]. With the estimated increase in health care expenses the expectations on health care technology are high.

Wearable technologies, wearable sensors, or simply wearables, can be described as smart electronic devices worn on the body as accessories or as part of the clothing [2]. The built-in sensors in wearables, such as accelerometers, gyroscopes, magnetometers and pressure and strain sensitive sensors can be used to quantify movements and body positions. When different electronic components are integrated into the fabric, enabling a garment to sense certain properties it is defined as an e-textile or a smart textile [8]. Accelerometers are most frequently used to quantify physical activity, gait related activity and upper extremity activity [9–12], but also to monitor and provide feedback on posture and arm movements during rehabilitation [13]. Algorithms developed to monitor motor fluctuation and medication evoked adverse symptoms in PD show promising results [14, 15]. In epilepsy, wrist-worn accelerometers have successfully been used to detect convulsive seizures by using machine learning techniques and trained decision classifiers [16, 17].

In addition to movements, physiological signs such as heart rate, respiratory rate, temperature, electrodermal activity and blood pressure, can be recorded using optical, electrophysiological and biochemical sensing [2]. Multimodal approaches that combine motion data with physiological signs have a potential to improve detection and differentiation of seizures in epilepsy [18]. Monitoring of autonomic alterations e.g. heart rate and blood pressure has also been suggested as means to improve diagnostics of non-motor symptoms in PD [19].

In recent years the development has progressed from sensors fastened on different body parts to sensors integrated into clothes [2, 13]. In the simplest application, the sensors are attached to the clothing for example using pockets or Velcro straps [20, 21]. In more embedded systems, the sensors are integrated into the fabric and connected by using coupling wires or conductive yarns [22–24]. However, research using sensors integrated into garments and clothing today comprises predominantly prototype design and evaluation in healthy populations [8]. The primary focus of these studies has been on movement or posture recognition [8]. Continuous monitoring and assessment of medical conditions by using smart garment technology is still in the initial stages of development and the usefulness in clinical practice is largely unknown.

User acceptance and preferences are critical components of usability and feasibility. Integration of new technologies in health care is dependent on whether patients and clinicians are willing and able to use them [1]. The recommendation that users need to be involved in the design and evaluation process of new technologies is well established [1]. Design concepts, such as Universal Design and Inclusive Design also have a clear focus on user involvement in the design process [25, 26] although the applicability and interaction between these design concepts in people with disabilities is an ongoing debate [27]. Despite the rapidly increasing use of wearable technology, the user acceptance and preferences for long term use in chronic neurological conditions is only sparsely covered [1]. Increased focus on these essential qualities is paramount to an efficient application of wearable technology in clinical practice.

This paper aimed to provide a step-wise description of the development process and evaluation of a wearable upper body garment with integrated sensors. The system is designed to allow monitoring of movements and physiological signs in order to detect and differentiate epileptic seizures, to monitor motor and non-motor fluctuations in PD and to quantify activity and upper limb function in stroke.

**Fig. 1:**
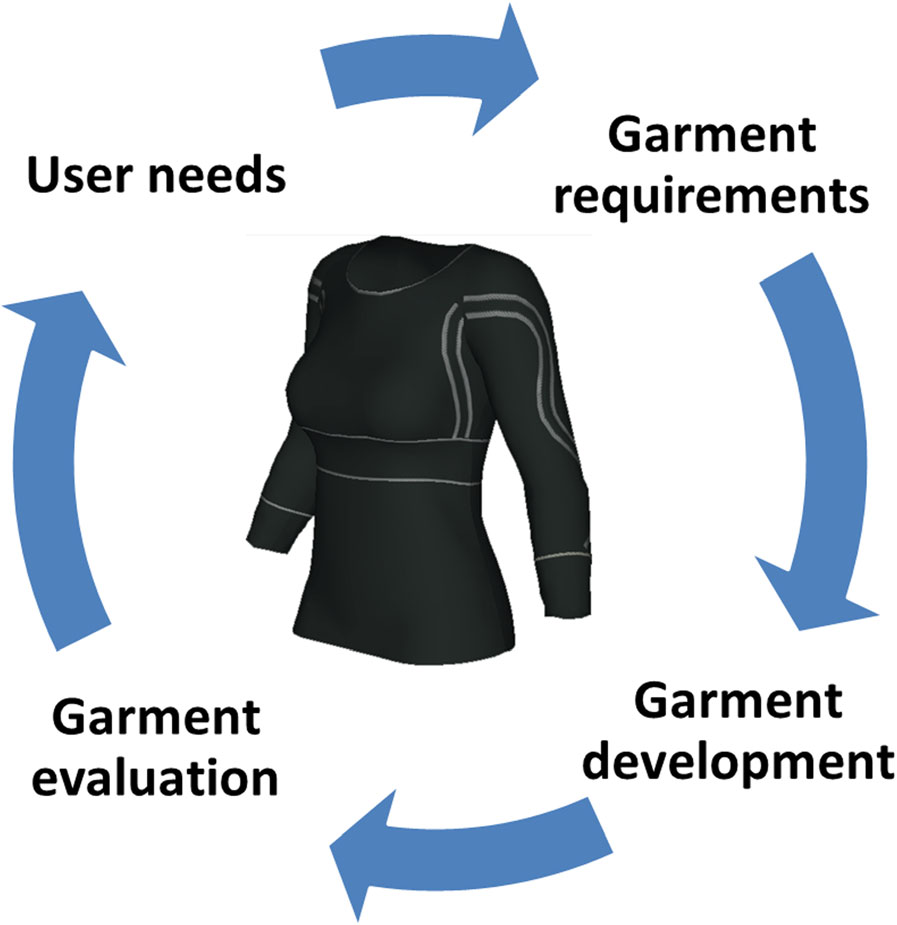
The iterative design development process

The development process is presented in four main steps: identification of user needs, specification of technical and garment requirements, garment development and production, and evaluation of garment design functionality and usability (Fig. 1).

## Results

### Step 1: identification of user needs

#### State-of-the-art overview

The findings from the mixed-methods systematic review [28] showed that accelerometer data from wrist worn sensors have mainly been studied to detect epileptic seizures and to quantify upper extremity activity and movement patterns in stroke and PD. Usability had only been evaluated in a handful of studies [28]. The readiness to use wearables was strongest for unnoticeable devices resembling ordinary items like watches and clothing, since the appearance in public was considered an important factor for willingness to use the device [29–33]. As expressed by the users, it was important that the sensors could be removed at any time [30]. In studies using straps to fasten the sensors, discomfort and difficulties to take the sensors on and off were described as problematic [29, 32]. For long-term use, sensors embedded into clothing or ordinary items were preferred to attaching the sensors directly on the skin [1, 33]. Other factors that were considered to influence the usability were comfort, how easy it was to take the wearable on and off, what the required wearing time was and whether the design was user friendly. Anticipated or experienced concerns included using the wearables incorrectly, confronting technical failures, and receiving insufficient support and feedback.

#### Clinical user needs

To monitor and characterize movements was the first priority for all three neurological conditions. Upper limb movements below the elbow along with trunk movements were considered central. Recordings of heart rate, heart rate variability, blood pressure, oxygen saturation and perspiration were considered important for better detection and differentiation of epileptic seizures and for monitoring of autonomic dysfunction in PD. Taking into account the technology readiness and shared priorities for all three conditions, it was concluded that movement data from arms and trunk together with heart rate was the first priority. Collection of data during free out of hospital everyday activities was highly prioritized, although validation of measurements in more controlled environments might be needed prior to free-living testing.

#### User perceptions of wearables

The focus group study revealed that simple, attractive, discreet design, comfortable to wear and water resistance were considered as facilitators of usability [34]. Aspects considered as barriers were: bulk, weight, and difficulty to take the garment on and off. Also the aspect of appearance was a barrier when the sensors were different from ordinary items or could not be covered when needed. Motor and cognitive disability was thought to hinder the usability when fine motor dexterity, memory and problem solving skills were required to handle the wearables. Complex systems were considered to increase the risk for using the wearables incorrectly or encountering technical problems. The potential benefits for improved treatment effect were, in general, valued more than the possible inconvenience of wearing the sensors. In addition to usability preferences, both patients and health professionals in the focus groups were confident that data from wearables can improve clinical decision making, but the health professionals also saw a potential risk for self-adjustment of medication. The use of wearables was, in general, perceived as cost-effective, but it was unclear by whom and how the data would be analysed and interpreted. It was a common understanding that privacy issues need to be addressed and it needs to be clear what is registered, why it is registered and how the output data will be used.

### Step 2: garment requirements

Based on the identified user needs it was considered important that the garment should be aesthetic and appealing to users and not cause stigmatization or unwanted attention. It should be safe to use and comply with personal integrity. The garment should allow use by people with disabilities and the design should be simple and intuitive. It should be easy to take on and off and it should not interfere with movements and common daily activities. The sensor components should be as small and light as possible.

The sensor zones around the torso and forearms needed to be tight to maximize skin contact and minimize motion artefacts. The sensors and garment needed to be robust, tolerate normal handling and washing using gentle machine wash cycle (the battery should be detached during washing). The time and effort for production needed to be reasonable. The results from the small pilot testing of fabric preferences showed that characteristics such as elasticity, breathability, not too warm, too cold, too thick or too thin were considered important. Some participants disliked the polyester fabric for everyday use and the color black was preferred. Men favored a v-neckline and women a more open u-neckline.

**Fig. 2:**
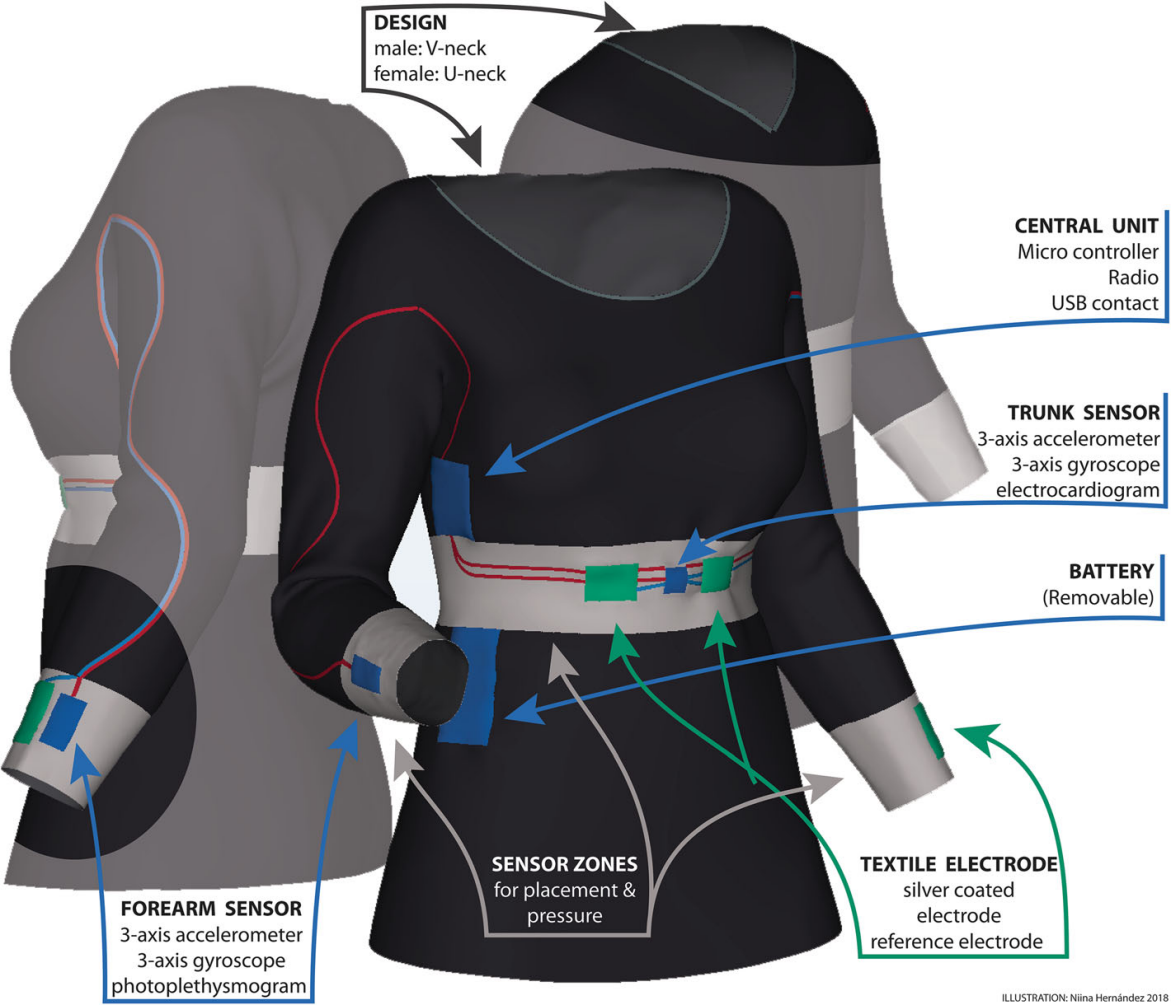
Schematic illustration of the garment design and components of the prototype

Movement data and heart rate (HR) were prioritized. A garment for the upper body with three measurement zones, one located centrally on the trunk and one on each forearm was designed (Fig. 2). The garment was designed with ¾ -length sleeves for two main reasons. First, the arm movements were considered important for detection of epileptic seizures as well as for quantification of dyskinesia in PD. The input from the distal part of the arm is also informative for quantification of the use of upper limb and symmetry quantification in stroke and PD. Second, the ¾ -length sleeve was preferred to long-sleeve since it would be more practical during the summer season and would be easier to use under other clothes.

### Step 3: garment development and production

#### Development of electronics

**Fig. 3:**
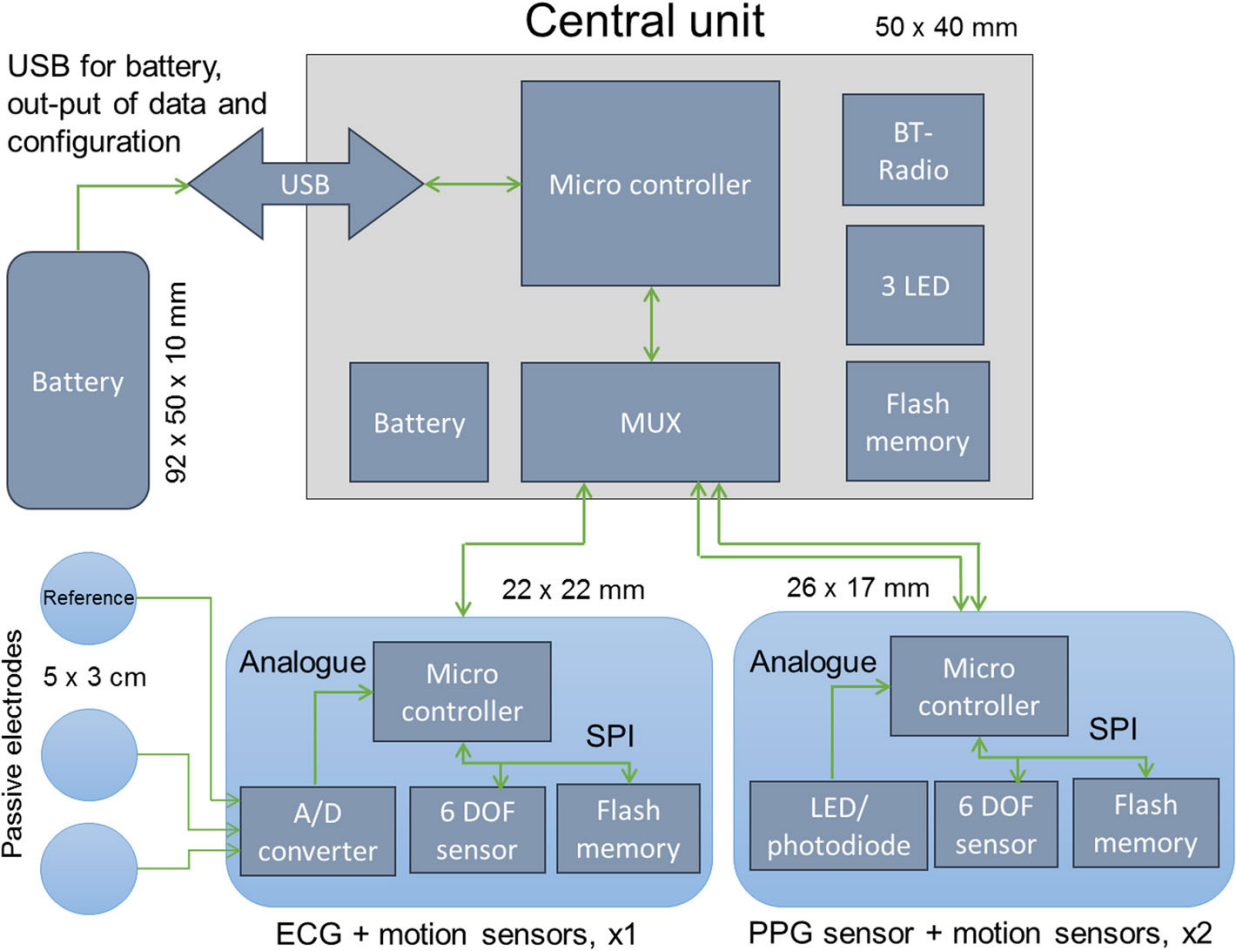
Schematic illustration of the system architecture

**Fig. 4:**
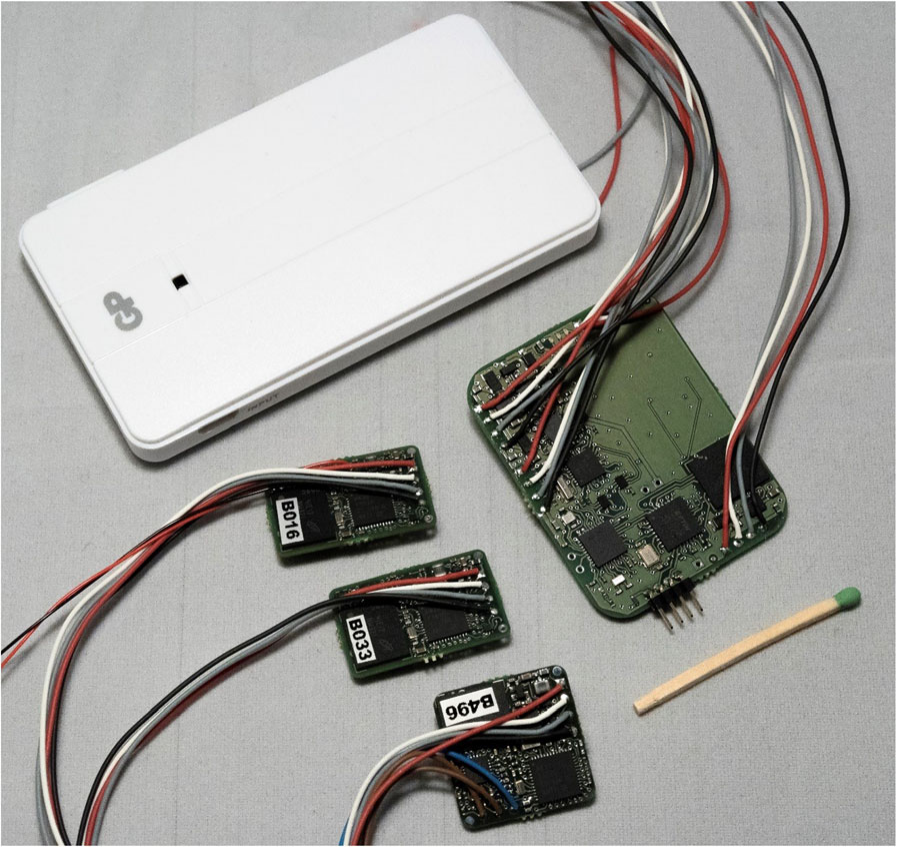
Components of the electronics: battery, central unit and 2 forearm and one trunk sensor unit

The electronics, sensor units and a central unit, consisting of conventional electronics were mounted on Printed Circuit Boards (PCB, Figs. 3 and 4). The sensor units on each arm’s sensor zone include a motion sensor (accelerometer and gyroscope) and an optical sensor (photoplethysmograph, PPG) for detection of pulse. The sensor units on the trunk sensor zone consist of a motion sensor and two textile silver coated electrodes for heart rate registration (electrocardiogram, ECG). The heart rate reference electrode is placed in the left arm’s sensor zone. All sensor units incorporate a flash memory and a microcontroller. The central unit consists of a microcontroller and a signal switching device (multiplexer, MUX), battery, flash memory, three LEDs and a low energy bluetooth unit. The central unit controls the measurement, communication and read out of data. An 1800 mAh battery was used that allowed approximately 24 h continuous recording at 100 Hz sampling rate, but individual configuration of the sensors including the sampling rate was possible. The start and stop of the measurement and the transfer of data required connection to a PC using a terminal application.

#### Development of garment and integration with electronics

**Fig. 5:**
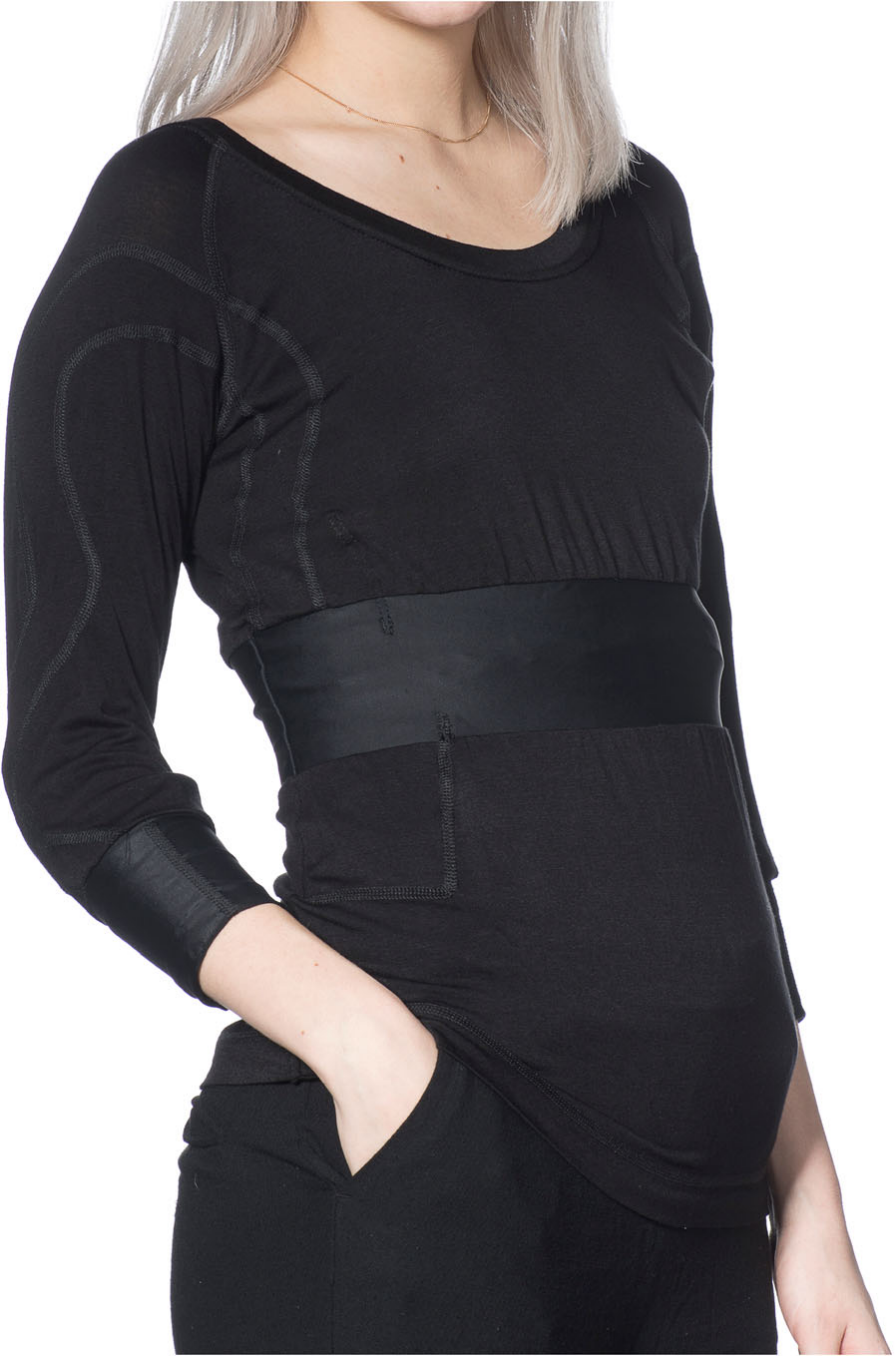
The first prototype of the garment with integrated sensors

First a small series of prototypes of viscose fabric with standard sizes for men and women was developed and produced at the Swedish School of Textiles. For the interconnections between sensors, the central unit and battery, a solution with coupling wires running into a channel with a meandering path between the sleeves and torso was used to allow unrestricted movements of the arm and trunk and to avoid mechanical strain on the interconnections (Figs. 3 and 5). The electronics were integrated into the garment during the sewing process, whereas the final connections to the central unit were carried out manually, after the assembly of the garment.

Subsequently, following iterative changes to the production process, a new series of 10 prototypes (5 for women, 5 for men) was produced. One important change was that the electronics were added in the final production step to reduce the risk of damaging the cables during sewing. The textile electrodes for heart rate were made from commercially available electrically conducting silver plated fabric (Shieldex), sewn into the garment over the chest and padded to add pressure to improve skin contact. Different technical solutions were tested to ensure that the electronics and connections were water resistant. For this prototype a transparent epoxy was used to seal the electronics. The transparency was necessary for the optical sensor to function, although the hardness of the epoxy was a risk to the cables. The textile electrodes and the electronics were connected with a thin tubing of conductive fabric (Shieldex) attached to the electrode during manufacturing. Part of the coupling wire from the electronics was wrapped around the tubing, and the connections were then secured and sealed with different dimensions of heat shrinking tubing. The weight of the prototype with all sensor components except the battery was 280 g.

#### Algorithm development

The parallel work with algorithm development made it possible to define the core features for each targeted neurological condition. In epilepsy, the accelerometer data from wrist sensors was used to develop machine learning algorithms based on standard binary classifiers in combination with a custom post-processing stage trained to discriminate the characteristic progression of tonic-clonic seizures. Evaluation showed that more complex non-linear classifiers (k-nearest neighbours, support vector machines and random forest) improved the algorithm generalizability and robustness for detection of epileptic seizures [35, 36]. In PD, both time and frequency domain representations of the accelerometer signal from wrist sensors formed the foundation for future development of algorithms quantifying movement-related signs, such as dyskinesia, bradykinesia and tremor. In stroke, several standard accelerometer features were evaluated as measures of activity levels. The measurement data indicated that the Signal Magnitude Area (SMA) and corresponding asymmetry indices (SMA ratio between more affected and less affected arm/leg) were most sensitive to variations in activity [37].

### Step 4: evaluation of garment design, functionality and usability

#### Evaluation of the garment functionality and washability

Garment functionality was evaluated in the first series of 10 prototypes (Fig. 5) by 14 healthy individuals who wore the garment for 2–48 h. The battery life-time for the system was approximately 12 h. The limited battery life-time was mainly caused by the LEDs in the two PPG sensors on the arms. An error in data storage on the memory flash card was identified as a critical problem and occurred in 8 out of 10 prototypes at some point after 1–3 measurement sessions. All electronic components passed the washing test, although in two cases the connection between the sensor and cables broke close to the hardened epoxy edge during washing or use. The textile silver plated electrodes conducted a clear electrical signal when slightly damp. As anticipated, the ECG and PPG signals were also sensitive to motion induced signal artefacts. The most robust signal was obtained from accelerometer and gyroscope sensors.

#### Evaluation of the usability and garment design

The experiences and perceptions of the users (individuals with PD and healthy adults) after wearing the garment for 2 days were analysed using qualitative content analysis methodology. Three main categories emerged from the interviews reflecting acceptance in daily life, concerns and worries and personal preferences.

Acceptance in daily life comprised acceptance for continuous use, acceptance in daily activities and acceptance for health and disease benefits. The garment was, in general, well accepted for continuous use over 2 days. Some participants expressed reluctance wearing the same garment for the next day after a night’s sleep. Taking a shower or washing in the morning was important to feel clean and fresh after a night’s use. Wearing time of 2 days was acceptable to most and a 4 to 14 days measurement was presumed to be acceptable if the garment could be changed or washed. A longer wearing time was considered meaningful when the results were important to one’s health or quality of life. Similarly, the aesthetic aspects, such as colour and design, were considered less important when the measurement would provide important information for disease management. The garment did not hamper movements during daily activities and participants in general felt comfortable when using it in front of other people. They described that wearing the garment was perceived as a positive experience and that others were interested in the technology and it was fun to talk about it. Many described that they forgot that they were wearing a special garment.

Concerns and worries reflected concerns with the garment fit and worries about electronics and technology. Participants described that it may be difficult to find a good fit that will suit people with different body sizes. Several participants perceived that the measurement zones (chest and arms) were too tight, especially when taking the garment on and off. Some reported that the tight arm cuffs pulled the neckline down too much. Most participants were aware of the battery, and a few found it troublesome at night. A smaller and lighter battery that could be changed without taking off the garment was a common wish. Half of the participants with PD received help with changing the battery and taking the garment on and off, but they also described that this was not different to the help they usually needed. Some participants worried that the electronics might break when the garment is taken on and off. They also felt unsure where they could get a solid grip when taking the garment on and off. Some participants reflected over the feeling of being monitored and that specific activities could be seen in the data. They also wanted to be able to get some indication on the garment, whether it is measuring or not. To see the results of the measurement was also of interest to many. Participants were conscious about the measurement in the beginning, but only a few commented that they were reflecting about the integrity and what can be seen in the data.

Personal preferences comprised aspects regarding comfort, style and use. Many described that the personal feeling of the garment was important. They found the garment to be comfortable, nice and pleasant. Men described that they felt unfamiliar with the neckline, which was perceived to be too wide and they also felt that it was unusual to use a ¾ long sleeve shirt under other clothes. To use a long-sleeve garment at night was also considered unusual by some. Black was preferred by most and the viscose material was perceived as pleasant. The breathability of the material and not feeling sweaty were described as pluses. The garment was predominantly used under other clothes and was not considered suitable for strenuous exercise when getting sweaty, during hot summer days or festive occasions.

#### Identification of improvements for the next prototype

The main improvement areas for the next prototype will include improved connection between electronics and cables to avoid disruption of the cables during use and improved water protection of the electronics and connections. The moulding of the electronics was done manually with clear epoxy resin. This process was time consuming and provided varying results. A more standardized and unified method will be used for encapsulating the electronic parts to ensure the washability and general robustness. Some minor changes in the hardware will be made, e.g. exchange of the flash memory cards, using smaller programming contacts and adding a magnetic switch for hardware reset. The design of the garment will also be upgraded with respect to comfort, design and robustness in accordance with the knowledge gained from the literature, focus groups and user interviews. The cables and connections will be strengthened to tolerate the pulling and strain of normal handling e.g. when taking the garment on and off. The location and design of the battery pocket will be altered, making it easier to change battery. A more elastic fabric will be used for the measurement zones and the neckline will be designed less wide. To ensure that the garment will be easy to take on and off by individuals with impairments, a changed design that allows loosening and tightening of the measurement zone over the chest will be implemented.

## Discussion

This paper provides a step-wise description of the initial design and development process of an upper body garment with embedded sensor technology designed to monitor movements and heart rate in people with epilepsy, PD or stroke. The first loop of this iterative design process described in this paper involved four main steps: identification of user needs, specification of technical and garment requirements, prototype development and production as well as evaluation of garment design, functionality and usability. The strengths of this project lay in the collaborative work of a multidisciplinary group of experts including medical, engineering, textile and material science organized in regular meetings, workshops and task groups.

The utilization of garments with integrated sensors for studying neurological conditions is in its initial stages. The expectations and potential are, however, high. Having access to continuous data rather than relying on patient narratives and clinical assessments is expected to provide new opportunities for clinical use, increase patient involvement and provide a more realistic picture of patients’ problems and functioning. In a more general perspective, the wearable sensor technology is believed to improve the quality of life for many patients to a reduced cost [1, 2]. Despite this considerable potential, there are still several challenges to be resolved.

Identification of user needs in targeted groups (patients and health professionals) is essential in the design and product development [1, 38]. In the current project, focus groups with patients and health professionals, qualitative analysis of the literature and work in multi-professional task groups and workshops were applied to form an informed decision process for the prototype development. Previous research in line with our results has established that users favour compact, preferably embedded and simple to operate devices [1]. The embedded quality implies that the user does not need to connect several parts and that the garment is easy to operate even for an untrained user. In addition, it should be possible to start and stop measurements and transfer data in a way that is simple for all potential users. This was also the starting point for the development of the current wearITmed system. For the first prototype, however, the start and stop of a measurement and the transfer of data required connection to a PC using terminal application, which is a limitation for a normal user. In addition, the change of battery was difficult to perform while wearing the garment due to the location and size of the battery pocket. These design issues will be considered in the next prototype.

The garment was in general well accepted and perceived as any ordinary clothing item. The users also preferred natural materials and a basic colour. Even when the aesthetic aspects matter, the focus groups and usability evaluation revealed that individuals with a neurological condition were less concerned about the aesthetics in terms of colour and design when they considered the measurement to be important and beneficial for their health or disease management. This emphasizes the advantage of using a regular looking garment for monitoring of movements or other physiological signs. The monitoring will become less in focus, and data gathered will reflect more closely the normal daily functioning and routine.

A central requirement for the current project was also that the garment needed to be easy to take on and off and easy to use by people with motor or cognitive disabilities. In consideration of this, for example, the neckline of the garment was designed relatively wide and the measurement zones close-fitting but elastic to ensure easy don and doff. Similarly, a regular size USB connection to the battery was selected to enable easier battery change for users with fine motor deficits. The neckline was, however, perceived too wide and the measurement zones needed to be even more elastic or adjustable. These observations will be used as input for the next prototype.

In the current project, the algorithm development was performed in parallel with the design of the garment prototype, which enabled a mutually informed design process. Statistical methods from the field of machine learning are in general well suited for detection of epileptic seizures [39] and for quantification of movement related signs common in PD [28, 40, 41]. Machine learning methods can be used to create objective models based on the observed data to assist clinical decision making. Large amounts of data with adequate variation are, however, required for sufficient explanatory power. This is an obstacle that might be overcome by improving informed selection of features to ensure that they are as sensitive and specific to the disease associated movement patterns as possible. To improve precision, patient-specific algorithms have also been suggested [42]. In this project, both the features and machine learning methods were carefully selected based on their properties and the application. Moving beyond the simplest linear classifiers to more complex non-linear classifiers may be needed to improve algorithm generalizability and robustness in the field of neurorehabilitation [36]. Although it is possible to bypass the “feature engineering” step using deep convolutional neural networks, it is currently unclear if such algorithms will pass regulatory requirements for medical decision support [43].

There have been several challenges in the collaborative work of the wearITmed consortium. In the current project, the embedded sensors were all commercial sensors connected by using coupling wires. This pragmatic approach was chosen to not delay the project and to get as early experience as possible from using a complete fully-functional prototype system. In the early stages of the project, sensor solutions using piezoelectric or conductive yarns were also considered, but postponed for future prototype development, since this technology was still in the experimental phase of development. The gap between our initial intention to use piezoelectric or conductive yarns and the state-of-the-art in terms of what can be delivered at present have also been corroborated in a recent review, which concluded that the current experiences and evidence is limited for the use of e-textiles or smart textile in neurological rehabilitation [8]. There is some evidence that e-textiles are able to detect large and slow movements, but due to the non-linear phenomena of hysteresis, the accuracy is still limited [8].

Considerable effort was invested to find a technical solution to ensure washability and robustness of the electrodes and interconnections. Our results show that the garment tolerated washing relatively well. For the next prototype, a more robust and durable solution will be developed for encapsulating the electronics and wire connections to ensure water resistance and robustness. The challenges with washability of garments with integrated sensors have been surprisingly little addressed in previous studies [8].

The wearable technology development has reached important milestones during the last decade. Despite this, the current technical solutions are not directly applicable for use in neurorehabilitation [1, 2, 8]. Increased collaboration between engineers, designers and clinicians is required to overcome this gap [1, 8]. Multidisciplinary initiatives, like the wearITmed consortium, are one way to increase awareness of and combine each other’s know-how to develop clinically relevant systems that meet patients’ needs.

A frequent experience is that it takes extra effort and time to find a common language and direction for a multidisciplinary project. This was also the case in this consortium. A further challenge is to involve patients in the design development. Although this typically takes extra effort, there is much to gain in terms of face value, relevance and general applicability of the developed product. However, care should be taken to keep the inconvenience and the extra strain in patients’ testings’ at a minimum, and to engage healthy volunteers for more general testing and evaluation of design elements and functionality. In addition, medical needs and real-world demands in clinical settings might not always match the technical solutions available or the developed solutions may not be practical in clinical practice. All these factors will influence the acceptance and success of wearable sensor technology in clinical practice. On top of these challenges, all the necessary regulations within the health care systems, requiring high level of safety, data security and evidence need to be considered early in the design process in order to not hamper the introduction of novel technologies.

## Conclusions

While there are still challenges remaining to be solved, the prototype developed by the wearITmed consortium is promising from both technical and user perspectives. Our results show that already after the first iteration of the development process, the garment prototype has potential to be used as a tool to monitor seizures in epilepsy, fluctuations in PD and activity levels in stroke. The future work of the wearITmed consortium includes two to three further iterations with a focus on garment design, algorithm development and exploration of the potential of including blood pressure and heart-rate variability monitoring in the targeted neurological conditions.

## Methods

The aim of this paper was to provide a step-wise description of the development process and evaluation of a wearable upper body garment with integrated sensors. The system was designed to allow monitoring of movements and physiological signs in order to detect and differentiate epileptic seizures, to monitor motor and non-motor fluctuations in PD and to quantify activity and upper limb function in stroke.

**Fig. 6:**
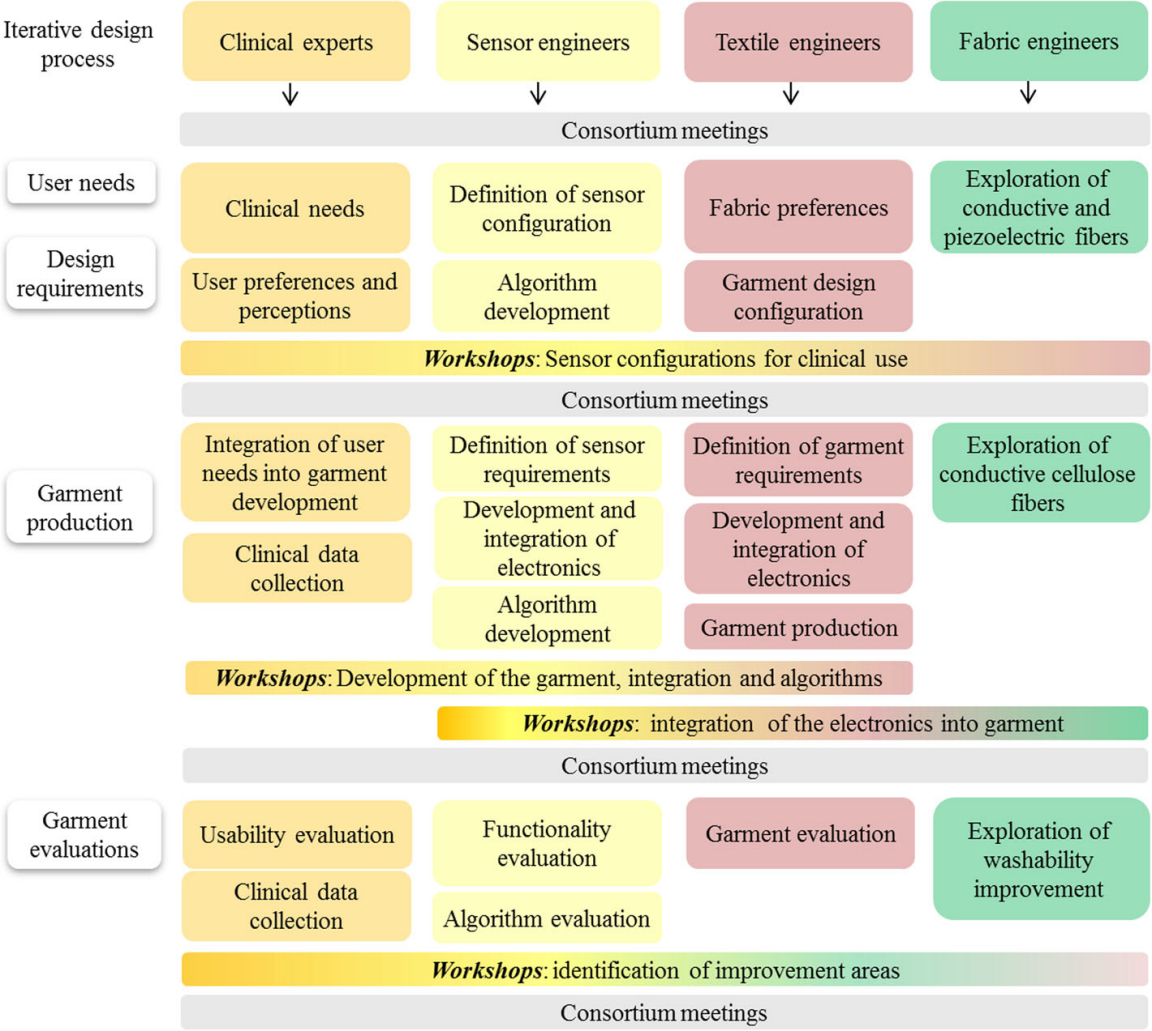
Organizational flowchart of the development process of the wearITmed consortium

The multidisciplinary wearITmed consortium was initiated in 2014 as a collaboration among researchers from medical, engineering, textile and material science. The consortium includes four main partners and the work has been performed through regular consortia meetings, work in task groups and hands-on workshops (Fig. 6).

The concepts of Universal Design and Inclusive Design were used to guide the conceptual part of the design process [25, 26]. The users in the current project were defined as people with epilepsy, PD or stroke as well as health professionals working with these conditions. The iterative development process embraces several loops each including four main steps: identification of user needs, specification of technical and garment requirements, garment development and production, and evaluation of garment design, functionality and usability (Fig. 1). In this paper the first loop of this iterative design process is described.

The parts of the project that included study participants were carried out in accordance with the declaration of Helsinki and ethical approval was received from the Regional Ethics Review Board in Gothenburg (507–15). All participants provided written informed consent prior to their participation in the project.

### Step 1: identification of user needs

#### State-of-the-art overview

A mixed-methods systematic review was conducted to synthesize knowledge and identify the main challenges from previous research using wearable sensors in epilepsy, PD or stroke [28]. The review aggregated knowledge both from quantitative and qualitative studies and identified: (i) how wearables have been used in different settings including laboratory, hospital and free-living setting, (ii) main challenges encountered using wearables regarding missing data and adherence, and (iii) how the wearables and their use were perceived by the potential or actual users. The results from this review were used as supplementary input for identification of user needs.

#### Clinical user needs

A separate task was conducted early in the process with the aim to define clinically relevant variables to register with wearable sensor technology for each neurological condition. Both movement-related and other physiological variables were considered. A priority list was set up that also included preferences for type of technology, where the sensors preferably should be placed on the body, and what kind of wearable garments would be realistic and most suitable for the clinical needs.

The main identified objectives for the use of wearable sensors in clinical settings were: (i) to detect and differentiate seizures in epilepsy, (ii) to quantify and monitor motor (bradykinesia, dyskinesia and tremor) and non-motor (heart rate and heart rate variability) fluctuations in PD, (iii) and to monitor exercise intensity and activity levels in stroke. Proven sensor technologies and commercially available components for the different parameters were prioritized. Results from this task formed the base for the definition of requirements specification described in Step 2.

#### User perceptions of wearables

A focus group study was conducted to explore perceptions regarding the use of wearable technology, including garments with integrated sensors, in disease monitoring and management as reported by individuals with epilepsy and PD as well as health professionals working with these patient groups [34]. The groups also discussed preferences regarding sensors, materials and aspects that facilitate or hinder the use of wearables. The results from the focus groups were used as guidance during the development process.

### Step 2: garment requirements

The garment specification task aimed to define the requirements for the design and sensor configurations in the first prototype. The priority lists generated during the task activity in Step 1 were discussed and conceptualized in a series of workshops. In this work the sensor modalities and configurations of interest for all three neurological conditions were prioritized.

In addition, an early informative pilot study with 3 men and 4 women of varying age and professional background was conducted to get input on preferences concerning the fabric that could be used for the prototype. The participants wore three different off-the-shelf sport t-shirts with different mix and thickness of polyester and polyamide fabrics over 1 day and night.

At early stages of the project, carbon filled conductive cellulose fibres and piezoelectric monofilaments were considered as sweat and heart rate sensors in the garment [44, 45]. This possibility was, however, postponed for future development, as the necessary technology was not readily available at the time.

### Step 3: garment development and production

The garment development and production included three main tasks: development of the electronics, development of the garment, and integration of the electronics into the garment. This was accomplished in an iterative process consisting of a series of workshops.

#### Algorithm development

Parallel to the development process of the garment prototype, potential sensor configurations were tested in all three targeted clinical populations. In this work, a set of 3-axial inertial motion sensor units (Shimmer 3, Shimmer, Dublin, Ireland) were used to gather accelerometer and gyro data from all targeted groups as well as from healthy controls in order to enable algorithm development and testing of the intended sensor configurations in the garment prototype.

In epilepsy, the algorithm development focused on detection of tonic-clonic seizures using accelerometer data from sensors mounted on both wrists of individuals undergoing video-EEG monitoring in a hospital setting. Machine learning and commonly used classification methods were used in the design and evaluation of algorithms for the detection of clinically meaningful features [36].

In PD, the algorithms focused on developing a set of parameters, derived from the accelerometer signal sensitive to variations in the motor symptoms bradykinesia, dyskinesia and tremor. A set of 5 sensors fastened on the torso, wrists and ankles was used and the data was collected while the person was performing common daily activities, e.g. making coffee, setting a table, doing dishes, taking a coat on and off and walking.

In stroke, the acceleration magnitude and the quantification of activity levels were of primary interest. As in PD, a set of 5 sensor units was attached on the torso, wrists and ankles. Data was collected during 48 h sessions during free-living activities [37].

### Step 4: evaluation of garment design, functionality and usability

#### Evaluation of functionality and washability

The functionality, i.e. the technical utility and washability, of the first prototype was evaluated in healthy individuals who wore the garment for 2–48 h. The garment was used during ordinary daily activities and at night when possible. The participants were asked to keep a log of their main activities e.g. taking a walk, sitting for longer periods, eating, sleeping as well as domestic, working, sports and leisure activities. The garment functionality was evaluated after use as well as after 40 degrees gentle machine wash and using air-drying or light centrifuge (400 rpm) and air-drying. The problems identified were recorded.

#### Evaluation of usability and garment design

The usability of the first prototype was evaluated through interviews both in healthy controls and in individuals with PD. Nine healthy adults (5 women, 4 men, age range 30–68 years) with varying professional background (engineering, health care, unemployed, and retired), and 6 individuals with PD (4 men, 2 women, age range 59–79) and their spouses (3 women and 1 man, age range 61–79) participated in the evaluation of the garment prototype and wore the garment for 24–48 h during ordinary daily life activities. In total 19 semi-structured interviews were performed. Participants were asked open question about their perceptions and experiences regarding the comfort during their daily activities and at night as well as their opinion of the measurement itself and the design of the garment. The interviews lasted about 30 min and were recorded and transcribed verbatim. A qualitative content analysis with an inductive approach was performed [46]. The meaning units were identified focusing on the manifest content close to the text and condensed into codes, which were sorted and abstracted into subcategories and categories. The coding and preliminary categorization of the first 5 interviews was independently performed by two researchers. The results were discussed and finalized by one researcher while keeping continuous discussions with the other researcher.

#### Identification of improvements for the next prototype

Information from the functionality and usability evaluation of the first garment prototype was evaluated in a series of workshops within the wearITmed consortium to identify common failures and define improvement areas and possible solutions for the next prototype.

## Abbreviations

A/D: Analog/digital
BT: Bluetooth
DOF: Degrees of freedom
ECG: Electrocardiogram
EEG: Electroencephalogram
g: Gram
HR: Heart rate
Hz: Hertz
LED: Light Emitting Diode
mAh: Milli-Ampere-hour
MUX: Multiplexer (signal switching device)
PC: Personal computer
PCB: Printed Circuit Boards
PD: Parkinson’s disease
PPG: Photoplethysmograph
rpm: Revolutions per minute
SMA: Signal Magnitude Area
SPI: Serial peripheral
